# Feasibility, safety and accuracy of a CT-guided robotic assistance for percutaneous needle placement in a swine liver model

**DOI:** 10.1038/s41598-021-84878-3

**Published:** 2021-03-04

**Authors:** Boris Guiu, Thierry De Baère, Guillaume Noel, Maxime Ronot

**Affiliations:** 1 Department of Radiology, St-Eloi University Hospital, 80 avenue Augustin Fliche, 34295 Montpellier, France; 2 Department of Interventional Radiology, Gustave Roussy Institut, Villejuif, France; 3 Department of Pre-Clinical, Biomedical and Analytical Investigations, Biovivo/Claude Bourgelat Institut, VetAgro Sup, Marcy l’Etoile, France; 4 Department of Radiology, Beaujon Hospital, APHP.Nord, Clichy, & Université de Paris, Paris, France

**Keywords:** Preclinical research, Medical imaging

## Abstract

Evaluate the feasibility, safety and accuracy of a CT-guided robotic assistance for percutaneous needle placement in the liver. Sixty-six fiducials were surgically inserted into the liver of ten swine and used as targets for needle insertions. All CT-scan acquisitions and robotically-assisted needle insertions were coordinated with breath motion using respiratory monitoring. Skin entry and target points were defined on planning CT-scan. Then, robotically-assisted insertions of 17G needles were performed either by experienced interventional radiologists or by a novice. Post-needle insertion CT-scans were acquired to assess accuracy (3D deviation, ie. distance from needle tip to predefined target) and safety. All needle insertions (43/43; median trajectory length = 83 mm (interquartile range [IQR] 72–105 mm) could be performed in one (n = 36) or two (n = 7) attempts (100% feasibility). Blinded evaluation showed an accuracy of 3.5 ± 1.3 mm. Accuracy did not differ between novice and experienced operators (3.7 ± 1.3 versus 3.4 ± 1.2 mm, *P* = 0.44). Neither trajectory angulation nor trajectory length significantly impacted accuracy. No complications were encountered. Needle insertion using the robotic device was shown feasible, safe and accurate in a swine liver model. Accuracy was influenced neither by the trajectory length nor by trajectory angulations nor by operator’s experience. A prospective human clinical trial is recruiting.

## Introduction

Ultrasonography (US) provides real-time guidance and does not require ionizing radiation, explaining that it is the main guidance modality for abdominal punctures^1,2^. However, US has many limitations compared to contrast-enhanced computed tomography (CT) and magnetic resonance imaging (MRI): less contrast resolution; difficulty in visualizing deep lesions; shadowing artefacts caused by gas, bone or bowel and increased inter-operator variability^3^. Tumors easily visible with CT or MRI may be inconspicuous (insufficient distinction in its echogenicity from surrounding liver tissue) or undetectable (located in an area inaccessible to US) with US^2^. This accounts for a large number of planned percutaneous tumor ablations (PTA) for hepatocellular carcinoma (HCC)^4^, and a drift towards palliative therapies in approximately 35% of patients with early stage HCC, as shown in real-life studies^5,6^.

CT fluoroscopy is mainly used within the axial plane because cranio-caudal off-plane insertions are more challenging. Consequently, access to tumors in the liver dome remains difficult with this technique because of lung interposition. Several techniques have been proposed to keep in-axial plane targeting^7–11^. Nevertheless, whatever the technique, such challenging tumor targeting frequently mandates multiple needle adjustments and intra-procedural imaging, thereby (a) prolonging procedure duration, (b) increasing radiation exposure to the patient and the staff, and (c) increasing the risk of complications. Among possible causes of needle misplacement, respiratory movements are one of the most important. During free breathing, the liver moves cranio-caudally, laterally and anteroposteriorly. Consequently, the accuracy of needle insertion also strongly depends on the management of respiratory movements.

Robotic technologies have been introduced to improve targeting accuracy regardless of operator experience, and to reduce radiation exposure to both patient and physician^3,12–14^. Provided accuracy is high, robotically-assisted needle placement could be very useful in overcoming the above-mentioned limitations of US and CT guidance.

A robotic device has been developed to allow safe one-shot needle insertion with CT guidance, so that the needle trajectory planned and insertion may be facilitated for physicians, especially for tumors in challenging locations. The robotic device provides a mean of 2 mm accuracy when evaluated in a phantom when targeting 8 mm radio-opaque spheres. Unlike the phantom experiment, targets located in an in-vivo model usually move during insertion. Therefore, the accuracy of robotic needle insertion must be evaluated in an animal model before any clinical use.

The aim of this study was to evaluate the feasibility, safety and accuracy of a CT-guided robotic assistance for in-vivo percutaneous needle placement in swine liver.

## Materials and methods

The study was conducted in compliance with EU Directive 2010/63/EU for animal experiments and with ARRIVE guidelines. The protocol of this study was submitted to the Ethics Committee of VetAgro Sup (French Ethical committee number 18) and authorized by the French ministry of higher education and research under Project number 1934—APAFIS# 21,179–2,019,050,211,163,818 v2).

### Animal model and preparation

Ten swine weighing approximately 50 kg were placed under general anesthesia and received pre-medication with Morphine at 0.1 mg/kg (subcutaneous route). Anesthesia was induced with Xylazine at 1.5 mg / kg (intramuscular route) and tiletamine + zolazepam at 3.75 mg / kg (intramuscular route). The animals were then intubated and placed under gas anesthesia (Isoflurane-O2). Injection of curare cisatracurium (0.1 to 0.4 mg/kg by intravenous route) was performed as many times as necessary, in order to constrain the breath movements of the animal. At least 5 (1 × 5 mm) fiducials (reference GTP1820 ≠ 2, Geotek Medical) were inserted by laparotomy in each animal, evenly spaced out in depth within a range of 60–120 mm. Fiducials were surgically inserted to make sure that the robot can target all fiducials wherever they are within the liver, and not just those that are percutaneously-implantable. At the end of surgery, the animals were awakened. On the day of the robotically-assisted procedures, the fiducials were numbered according to their CT-scan position.

### Percutaneous robotically-assisted needle insertions

#### Robotic device

**Figure 1:**
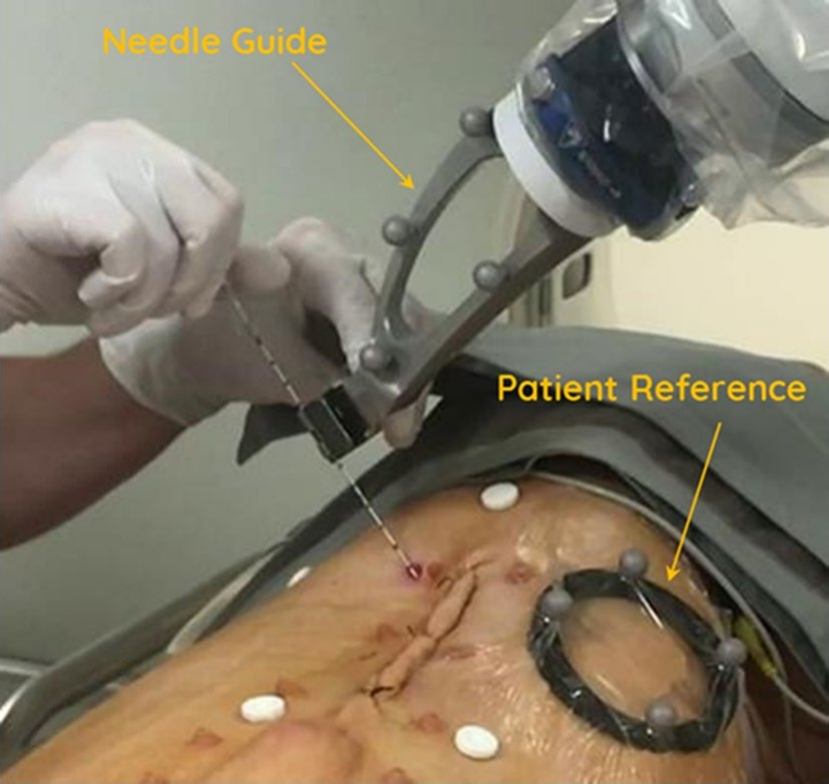
Example of a robotically-assisted needle insertion.

The robotic device, a prototype of Quantum Surgical SAS (Montpellier, France) produced for the preclinical study on animals, is an image-guided device that assists the physician during CT-guided procedures (Fig. 1). It allows to plan the insertion of needles on medical images and provides accurate mechanical guidance in accordance with the planning.

**Figure 2:**
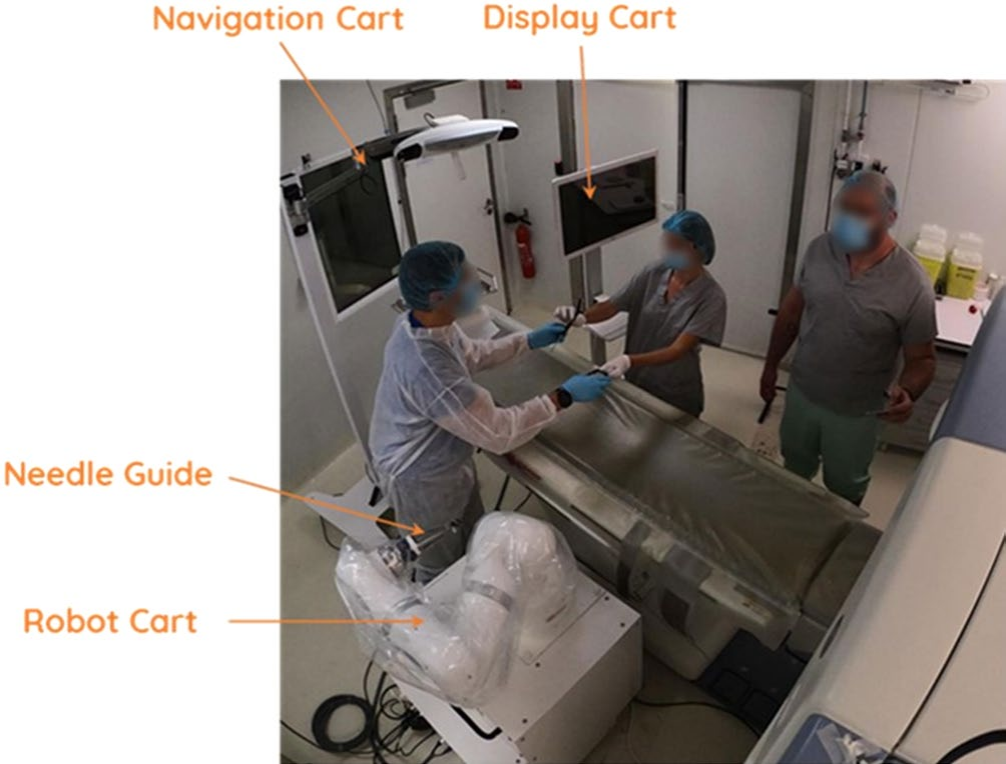
Components of the robotic device. The device is composed of a mobile robot, navigation and display carts. Needle guide is attached to the robot arm and provides mechanical guidance for rigid straight needles. Patient reference (with 4 embedded radiopaque markers) is adhesively attached on to the patient’s skin and enables to monitor respiratory motion.

The robotic device consists of the following main components:

- A mobile *robot cart* (Quantum Surgical, Montpellier, France) positioned next to the patient, which carries a robotic arm.
- A mobile *display cart* (Quantum Surgical, Montpellier, France) positioned next to the operator, which carries a touch screen.
- A mobile *navigation cart* positioned next to the table, which carries a navigation camera (NDI Polaris Vega, Ontario, Canada). The optical tracking camera allows spatial tracking of the needle guide and patient reference.
- A *needle guide* (Quantum Surgical, Montpellier, France) attached to the robotic arm, which provides mechanical guidance of instruments. The needle guide is localized by the navigation camera using six disposable reflective spheres.
- A *patient reference* (Quantum Surgical, Montpellier, France) attached to the patient’s skin, which monitors the patient’s motions including breathing. The patient reference can be identified on the CT images with four embedded radio-opaque markers (Fig. 2) and is localized by the navigation camera using four disposable reflective spheres.

The software (Quantum Surgical, Montpellier, France) running on the robotic device enables the physician to review medical images, plan a needle trajectory towards a target through the skin, monitor respiratory motion, and automatically position the needle guide according to the plan.

**Figure 3:**
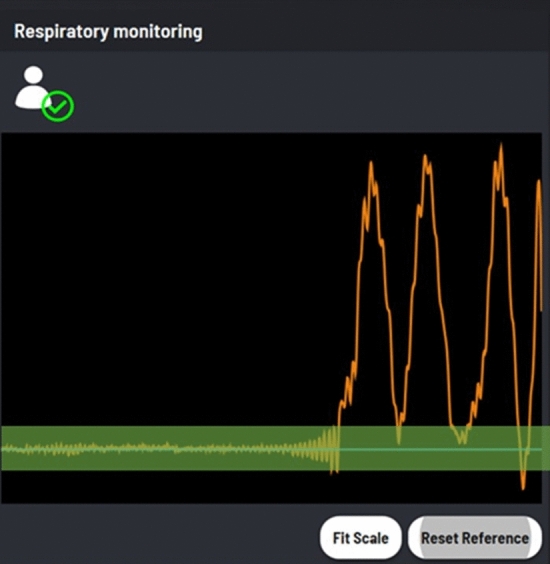
Screenshot of the respiratory monitoring function (home-made software). The orange line shows the live respiratory movement. When an apnea is performed, the curve stabilizes and a reference level can be defined (blue line). A gating threshold is also displayed as a green tube to help verifying apnea or breath-hold repeatability.

The proprietary respiratory monitoring module (Quantum Surgical, Montpellier, France) enables real-time tracking & display of respiratory cycle, and recording & display of a respiratory reference level to coordinate robotically-assisted needle insertions with CT-scan acquisition. The real-time curve representing the breathing cycle (Fig. 3) allows the operator to visually check the good repeatability of apnea and to record a breathing reference level. The extraction of the breathing curve is performed by a 3D motion analysis of the patient reference.

The device provides a 2 mm accuracy when evaluated in vitro (abdominal phantom). The registration between the actual patient position on the table and the CT images is automatically performed: the optical tracking camera detects the position of the patient reference (with reflective spheres) in space whereas the markers of the patient reference (ie, embedded radio-opaque markers) are located in the CT images by the software. The registration between the robotic system and the patient is performed using the positions of patient reference and robot arm both located by the optical tracking camera owing to the reflective spheres (on both the patient reference and the needle guide).

#### Apnea repeatability test

To verify that the liver is repositioned at the same location under repeated apneas, an apnea repeatability test has been performed on the 10 swine, using the respiratory monitoring module. Apnea was induced during all CT scan acquisitions and robotically-assisted needle insertions by turning off the ventilator at end-expiration. In addition, curare cisatracurium (0.1–0.4 mg/kg IV) was injected as many times as necessary in order to constrain the breath movements of the animals.

Two successive unenhanced CT-scans were acquired during apnea to assess their repeatability. In-between, animal breathing was resumed. The location of each fiducial (DICOM coordinates) was determined on the two CT-scans, using standard DICOM image viewer software (Myrian v2.4, Intrasense, Montpellier, France; https://intrasense.fr/fr/). The coordinates of each fiducial relative to the patient reference were determined and the 3D Euclidean deviation between the two acquisitions was computed to determine the displacement of the fiducial under repeated apnea. The apneas were considered as repeatable if the displacements of all fiducials between the two CT scans were below 2 mm.

#### Imaging

All CT scans were performed using a 16-slice Brightspeed CT scanner (General Electric). Animals were positioned supine and acquisitions were centered on the abdomen with a 35 cm field of view. The scanning parameters were 120 kV, 150 mA, 0.8 s gantry rotation time, and 0.938 pitch. Scans were reconstructed with a 0.625 mm slice thickness using a standard soft tissue convolution kernel.

#### Needle trajectory planning

**Figure 4:**
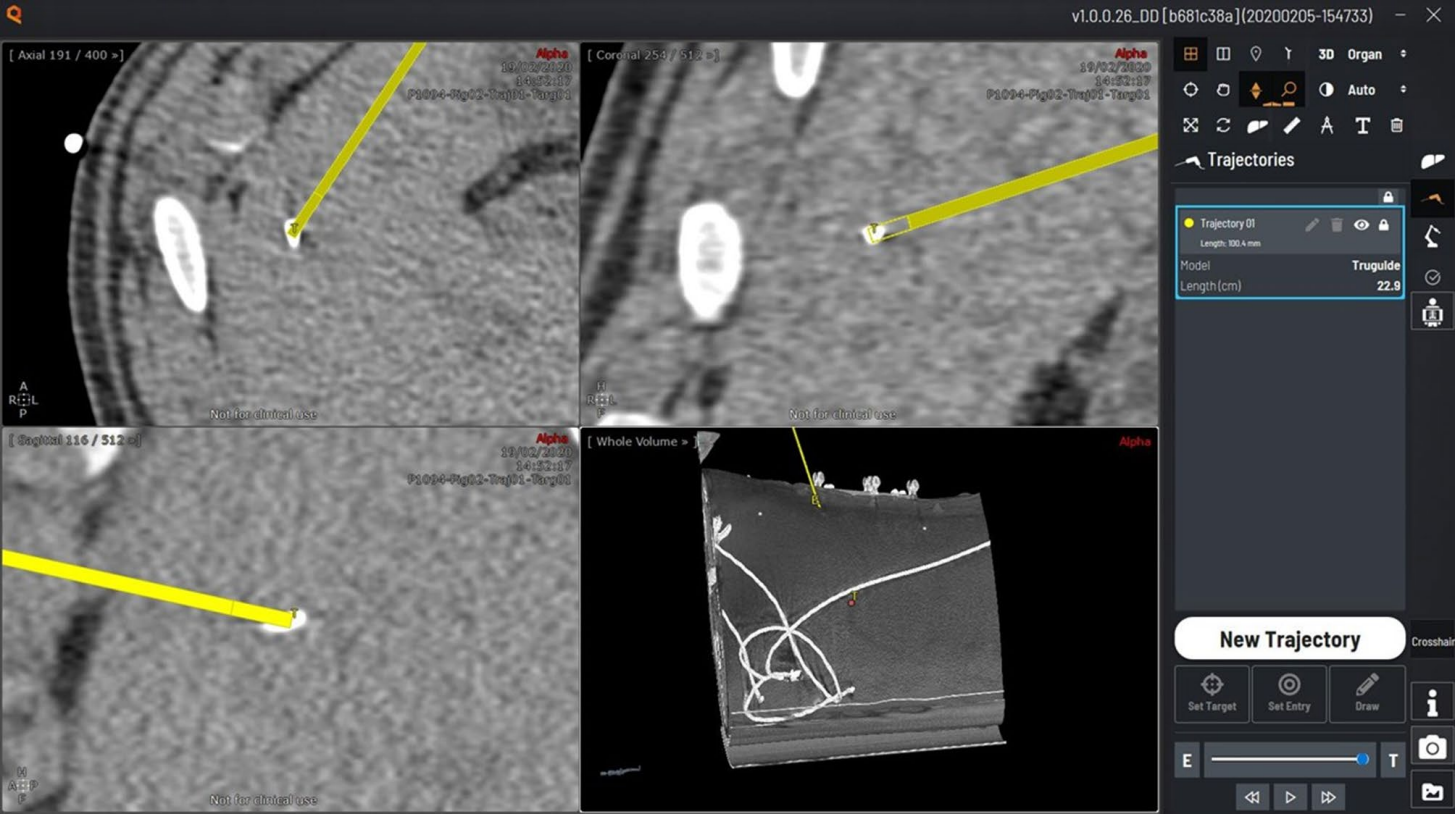
Screenshot of the planning software (home-made software). The needle trajectory (in yellow) is planned by defining the entry (skin) and the target points.

A pre-interventional image was acquired under apnea and then loaded in the software. The needle trajectory was defined with the planning software (Fig. 4). The choice of targeted fiducials was left to the operators. The target was defined at the center of a fiducial marker. The skin entry point and needle pathway were reviewed to avoid bones and critical structures in the abdomen. The needle trajectory length (distance between target point and skin entry point) was recorded.

#### Robotically-assisted needle insertion

The procedures were performed by one experienced IR (among three with at least 10 years of experience in image-guided procedures) or by a veterinarian (with no experience in image-guided procedures) considered as a novice. The robot arm was sent to its predefined HOME position. The needle guide was attached to the robot arm. Then the software automatically located the patient reference markers in the images. The position of each marker was verified and manually adjusted whenever necessary.

A 17G disposable Coaxial Biopsy Needle (Bard Medical, Covington, USA) with the appropriate needle length (from 10.6 to 20.8 cm), i.e. a needle with a length slightly greater than the trajectory length, was determined and selected into the planning software.

The robot arm was manually pre-positioned close to the animal’s abdomen using a free hand-guiding mode. Then, the robot arm automatically positioned the needle guide in line with the planned trajectory. An incision was made in the skin at the needle entry site prior to needle insertion. Then, the operator inserted the needle through the needle guide until the end stop was reached. The needle was then released from the needle guide and the robot arm was manually withdrawn away from the entry site.

#### Needle placement verification

A CT-scan image was acquired under apnea to assess needle placement accuracy. The procedure was deemed complete when the operator judged the needle tip as being ≤ 5 mm of the target. Otherwise, an additional attempt was allowed with the same planning. A third attempt was not permitted.

#### Safety assessment

Five minutes after needle removal, a contrast-enhanced CT-scan of the abdomen was performed within 60 s after injection of 30 mL of ioxehol (Omnipaque, 350 mg/mL) at a rate of 2.2 mL/s. Any procedure-related complications were noted by the operator.

### Study endpoints

Primary endpoints were needle placement accuracy and safety. Needle placement accuracy (3D deviation) was defined as the distance between the needle tip and the center of the targeted fiducial, measured on CT-scan image immediately after completion of each needle insertion. Needle placement safety corresponded to the description of any procedure-related complications occurrence on post-intervention contrast-enhanced CT-scan.

Secondary endpoints were needle placement feasibility and adjustment. Needle placement feasibility was the number/percentage of successful insertions (ie., when the needle tip was deemed < 5 mm to the target and no problem occurred with the robotic device during the intervention). Needle placement adjustment was defined as the number/percentage of needle replacement using the robotic system to reach the target. Only one additional attempt was permitted using the same planning as the first attempt, when the needle tip-target distance was estimated to be > 5 mm.

### Statistical analysis

For this pilot study, no specific sample size calculations were performed. Indeed, the inclusion of ten animals was considered sufficient to assess, and cope with, inter-subject variability and to meet logistical capabilities. In addition, at least three percutaneous CT-guided needle insertions per animal and a total of at least 35 CT-guided needle insertions were expected based on literature data^12,13,15^.

Categorical variables are presented using frequency and percentages, while continuous variables are presented using frequency of missing, non-missing, mean ± standard deviation, median [minimum and maximum].

**Figure 5:**
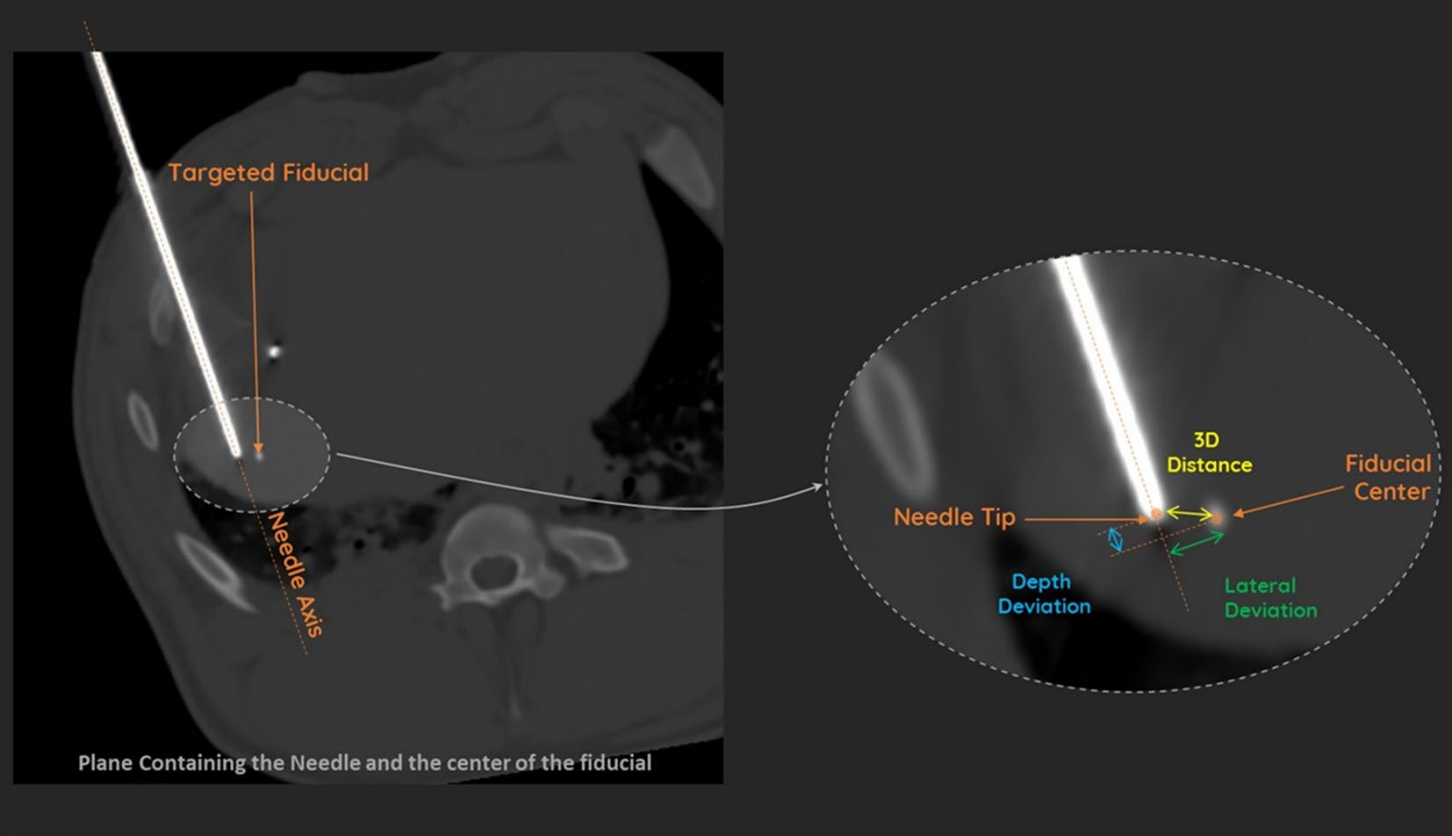
Measurement of 3D (Euclidian), lateral and depth deviations (Myrian software, Intrasens, Montpellier, France).

The needle placement accuracy (3D deviation), lateral deviation and depth deviation were described as continuous variables. All measures were performed by an experienced IR (blind review). The needle placement accuracy was determined by computing the 3D (Euclidean) deviation between the actual needle tip position and the center of the fiducial (targeted point). The DICOM Image viewer was used to get points coordinates in DICOM images and to evaluate 3D deviation, lateral deviation and depth deviation (Fig. 5). The needle placement accuracy was evaluated on at least 3 fiducials per animal.

3D deviation least-squares mean estimates (and 95% upper limit) were computed using a linear mixed model including random effects for animal and fiducial (nested in an animal) and adjusted on the operator experience ‘novice versus experienced operator’ (fixed effect). *P*-values testing for inferiority to 5 mm were produced.

Correlation between, respectively, 3D deviation and skin-target distance, orbital and cranio-caudal angulations were investigated using Pearson correlation coefficient. All statistical analyses were produced using SAS 9.4 software (Cary, NC).

### Human and animal rights

The study was performed in animals and carried out in compliance with the ARRIVE guidelines.

## Results

### Percutaneous robotically-guided needle insertions

**Figure 6:**
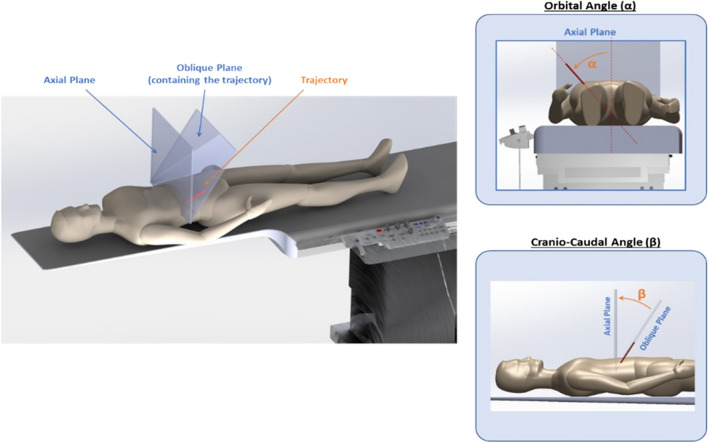
Axial and coronal maximum intensity projection CT images showing the placement of fiducials in a swine live.

A total of 66 fiducials were surgically implanted in 10 swine and 64 deeply spaced fiducials were identified on CT-scan control images (five, six or seven fiducials per animal inserted into the liver) (Fig. 6). A total of 33 fiducials were targeted during the study by the four operators (three experienced and one novice). A total of three fiducials were targeted twice in three animals, each with different angulations. The remaining thirty fiducials were targeted once.

A total of 43 needles were inserted, 36 of which were inserted at first attempt (tip-target distance was estimated to be < 5 mm) and 7 required a second attempt (tip-target distance was considered > 5 mm). No technical failure was reported.

**Figure 7:**
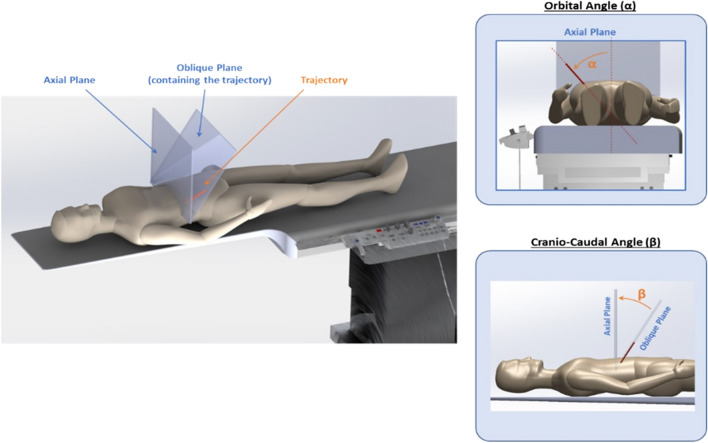
Definition of orbital and cranio-caudal angulations.

For the 36 needles placement estimated at < 5 mm from the target after the first attempt, the novice planned 16 (44%) trajectories on 5 animals and each experienced IR planned between 6 and 7 trajectories on 2 animals, with a total of 20 trajectories (56%). The median trajectory length was 83 mm (IQR 72–105 mm, range 47–117 mm); the median orbital angulation was + 2.6° (IQR − 3.6 to + 28°, range − 37° to + 55°); and the median craniocaudal angulation was + 10.5° (IQR + 0.4 to + 26.5°, range − 2.3° to + 61°) (Fig. 7). The median time between the beginning of the procedure (robotic device switched on) and the last needle placement assessment (needle in place) was 22 min (IQR 18–25.5 min, range 15–45 min) for novice, 25 min (IQR 19.5–32 min, range 15–41 min) for experienced IRs and 24 min (IQR 18–29.5 min, range 15–45 min) for all operators (Table 1).

**Table 1:** Characteristics of percutaneous robotically-assisted needle insertions.

Parameter	N	Mean (SD)	Median (Q1; Q3)	Min; max
Distance from skin entry point to target (mm)	36	86.5 (19.7)	83.4 (72.3; 105.0)	47.4; 117.0
Orbital angulations (°)	36	9.6 (23.0)	2.6 (− 3.6; 27.6)	− 36.8; 55.2
Cranio-caudal angulations (°)	36	14.1 (15.5)	10.5 (0.4; 26.5)	2.3; 61.0
Time from the beginning of the procedure (switch on the device) to last needle placement (needle in place)-all operators (minutes)	36	24.8 (7.6)	24.0 (18.0; 29.5)	15; 45.0
Time from the beginning of the procedure (switch on the device) to last needle placement (needle in place)-experienced operators (minutes)	20	26.3 (7.6)	25.0 (19.5; 32.0)	15; 41.0
Time from the beginning of the procedure (switch on the device) to last needle placement (needle in place)-novice operator (minutes)	16	23.0 (7.3)	22.0 (18.0; 25.5)	15; 45.0

Analyses based on last needle insertion.

### Assessment of apnea repeatability

Two apnea tests were conducted for each animal. Per-fiducial displacement comparison showed that all 64 fiducials depicted on CT moved less than 2 mm between two consecutive apneas. The 3D deviation of the fiducials between CT-scans acquired during two consecutive apneas was significantly lower than 2 mm (*P* < 0.0001) with a least-squares mean (and 95% upper limit) of 0.61 mm (0.77).

### Needle placement feasibility and adjustment

All needle insertions (100%, 43/43) were successful (no problems occurred with any of the insertions).

On the 36 needle insertions estimated to be < 5 mm from the target after the first attempt, only 7 (19.4%) required a needle placement adjustment (i.e., the need for a second insertion on the same planning because the first 3D deviation was considered > 5 mm).

### Needle placement accuracy and safety

When considering all needle insertions, 33/43 (76.7%) had a 3D deviation lower than 5 mm, with a mean lateral and depth deviation of 3.3 ± 1.8 mm and 1.3 ± 2.6 mm, respectively. The mean 3D deviation was 4.2 ± 2.0 mm and was found significantly lower than 5 mm (LS mean estimate [95% upper limit]: 4.1 [4.7], *P* = 0.014). When comparing 3D deviation between novice and experienced operators, the mean 3D deviation of all needle insertions was 4.0 ± 1.6 mm and 4.3 ± 2.3 mm, respectively. The novice inserted 14/18 (77.8%) needles with a 3D deviation lower than 5 mm, and the IRs inserted 19/25 (76%) needles with a 3D deviation lower than 5 mm (p = NS).

When considering the final needle insertions for each procedure, 33/36 (91.7%) had a 3D deviation lower than 5 mm, with a mean lateral and depth deviation of 2.8 ± 1.2 mm and 1.1 ± 2.1 mm, respectively. The mean 3D deviation was 3.5 ± 1.3 mm and was found significantly lower than 5 mm (LS mean estimate [95% upper limit]: 3.6 [4.0], *P* < 0.0001). When comparing the 3D deviation between novice and experienced operators, the mean 3D deviation of the last needle insertions was 3.7 ± 1.3 mm and 3.4 ± 1.2 mm, respectively (*P* = 0.44). The novice inserted 14/16 (87.5%) needles with a 3D deviation lower than 5 mm, and the experienced IRs inserted 19/20 (95%) needles with a 3D deviation lower than 5 mm (p = NS). The results of needle placement accuracy are presented in Tables 2 and 3.

**Table 2:** Needle placement accuracy (in mm)—Summary table of 3D, lateral and depth deviations-All operators.

Parameter	N	Mean (SD)	Median (Q1 ; Q3)	Min ; max	LSmean [95% upper limit]	*P* value*
3D deviation (mm)	43	4.19 (2.03)	3.70 (2.70 ; 5.30)	0.80 ; 10.20	4.15 [0 ; 4.74]	*P* = 0.0138
	36	3.50 (1.25)	3.40 (2.60 ; 4.35)	0.80 ; 5.90	3.59 [0 ; 3.99]	*P* ≤ .0001
Lateral deviation (mm)	43	3.28 (1.80)	3.10 (2.00 ; 4.10)	0.60 ; 8.90	3.26 [0 ; 3.83]	*P* = 0.0005
	36	2.82 (1.18)	2.75 (1.85 ; 3.65)	0.60 ; 5.00	2.88 [0 ; 3.32]	*P* ≤ .0001
Depth deviation (mm)	43	1.27 (2.62)	1.5 (0.00 ; 2.70)	-5.00 ; 7.20	1.22 [0 ; 2.04]	*P* ≤ .0001
	36	1.07 (2.06)	1.40 (0.10 ; 2.20)	-3.80 ; 6.20	1.05 [0 ; 1.86]	*P* ≤ .0001

Analyses based on all or last needle insertion measure.

*P value for unilateral testing distance H0: > 5 mm vsersu H1: ≤ 5 mm.

Least Square (LS) Mean and 95% Confidence Interval (CI) taking into account intra-animal and fiducial nested in animal variability structure.

**Table 3:** Needle placement accuracy (in mm)—Summary table of 3D, lateral and depth deviations—By operator.

Parameter		N	Mean (SD)	Median (Q1 ; Q3)	Min ; max	LSmean [95% upper limit]		*P* value*	Effect of type of operator
3D deviation (mm)									
Novice operator		16	3.69 (1.31)	3.40 (2.75 ; 4.95)	1.50 ; 5.90				
Experienced operators		20	3.35 (1.22)	3.30 (2.60 ; 4.10)	0.80 ; 5.40				
						3.62 [0 ; 4.04]		*P* = 0.0003	0.44
Lateral deviation (mm)									
Novice operator		16	2.98 (1.25)	3.40 (1.90 ; 4.00)	0.70 ; 5.00				
Experienced operators		20	2.69 (1.14)	2.50 (1.80 ; 3.35)	0.60 ; 5.00				
						2.90 [0 ; 3.37]		*P* ≤ .0001	0.57
Depth deviation (mm)									
Novice operator		16	1.14 (2.40)	0.90 (-0.05 ; 2.35)	-3.10 ; 6.20				
Experienced operators		20	1.02 (1.81)	1.50 (0.40 ; 2.20)	-3.80 ; 3.30				
						1.09 [0 ; 2.00]		*P* ≤ .0001	0.65

Analyses based on last needle insertion measure.

*P value for unilateral testing distance H0: > 5 mm versus H1: ≤ 5 mm.

Least Square (LS) Mean and 95% Confidence Interval (CI) taking into account type of operator in fixed effect, intra-animal and fiducial nested in animal variability structure.

No procedure-related complications were observed on post-procedural CT-scan.

### Impact of trajectory angulation and length

**Figure 8:**
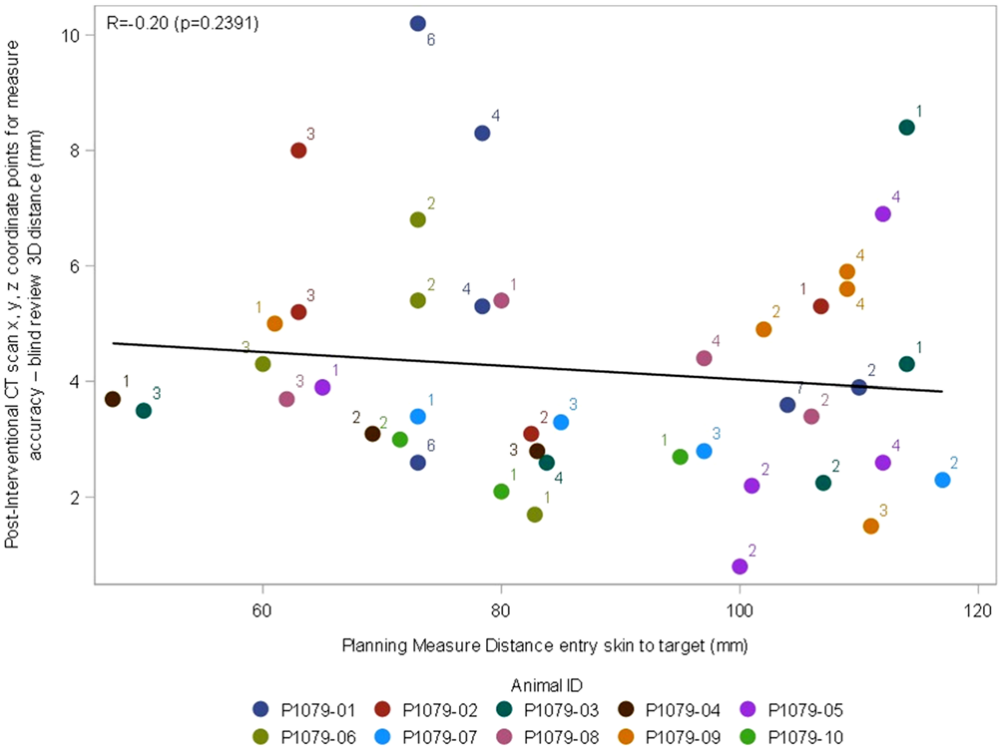
Correlation between 3D (Euclidian) deviation and skin-to-target distance.

Orbital or craniocaudal angulations of the needle insertion did not impact needle placement accuracy (r = 0.2; *P* = 0.24 and r = 0.05; *P* = 0.78 respectively) and had no effect on the number of attempts (*P* = 0.53 and *P* = 0.37, respectively). No correlation was found between needle placement accuracy and trajectory length (= − 0.2; *P* = 0.24) (Fig. 8).

## Discussion

The mean accuracy of 3.5 mm we report here is among the best results reported for robotically-inserted needles in in-vivo experiments (usually ranging between 2.7 and 10.2 mm ^12,13,16,17^) and is clinically more than acceptable. Indeed, less than 5 mm targeting error is a pre-requisite for a successful biopsy of a 10 mm lesion^13^. In most papers on robotic needle insertions, assessment of insertion accuracy according to needle tract angle is lacking, and cranio-caudal angulations are usually lower, when reported^3^. The mean needle placement accuracy was impacted neither by the trajectory length nor by the orbital or cranio-caudal angulations of trajectories, despite a wide range of values. Five of the 36 (14%) needle insertions had a cranio-caudal angle outside the (− 30° to + 30°) range, usually permitted by CT gantry tilting. These data reflect the wide flexibility of the system (6 degrees of freedom). The location of the robot out of the bore of the CT gantry further facilitates the procedures in large patients and makes lateral needle access possible.

Among all possible causes of needle misplacement, respiratory movements are one of the most important. During free breathing, the liver moves 20 mm cranio-caudally, about 5 mm laterally and 10 mm anteroposteriorly^18,19^. Accurate and repeatable management of respiratory movements is therefore mandatory to improve the accuracy of needle insertion. Respiratory movements are controlled by using mechanical ventilation and muscle relaxants^3,20^. The system was able to monitor breathing movements (and thus, target movements) in real-time and showed that the repeatability of apnea was excellent (almost negligible deviation at 0.6 mm) when both stopping the ventilation at end expiration and using curare.

Other sources of needle deviation include the intrinsic accuracy of the system, needle bending and liver deformation during needle insertion. During needle insertion, a peak in force is observed when puncturing the liver capsule followed by a sharp decrease and subsequent variations due to friction, internal stiffness, and cutting forces as well as collisions with, and puncture of, internal structures^21^. System stability ensured by the robotic arm and rapid needle insertion enabled by needle guide certainly contributed to the high accuracy. Rapidity is useful to reduce deformation of liver tissue and displacement of insertion site (reported to range between 9 and 13 mm depending on needle type^13,22^) observed when the needle is inserted slowly^22^. Moreover, due to the design of the needle guide and systematic skin incision at the entry site, no needle bending was observed. Some robotic systems are designed to compensate for target motion during needle insertion using per-procedural imaging checkpoints^13,16,20^. They have shown high accuracy in the liver (2.7–3.4 mm) but at the expense of increased patient radiation exposure and procedure time due to multiple CT controls. Another limitation is the constraint on needle access and possible trajectories because the robot is basically in the area of the CT-gantry. Such robots often lack force (haptic) feedback despite the fact that such feedback offers safety benefits in manual insertions^22^. Finally, the needle connected to the robot during the whole insertion procedure might cause damage in case of unexpected patient movements. This was not the case here since the robot was located outside the CT-gantry, and because the needle was quickly detachable from the needle guide.

In vivo studies on robotic needle insertions reported, in addition to high accuracy, a decreased number of needle adjustments as compared to manual insertion^12,17^. In our pilot study, only 19% needle placement adjustments were necessary to reach 91.7% of procedures with an accuracy of < 5 mm. Reduction of needle manipulations certainly reduces the risk of puncture-related complications, especially bleeding^12^.

A decrease in accuracy was reported when needle insertion was performed manually under CT guidance by novice operators^23,24^. One of the key advantages of robotic platforms is to reduce the influence of experience. Very few studies reported on inter-physician outcome comparison^12,16^ and we found only one study where two operators had different levels of IR experience^16^. Here we were able to compare the accuracy of needle insertions performed by three experienced IRs (with at least 10 years of experience in liver IR) and by a veterinarian who was completely novice in image-guided needle insertions. Interestingly, the accuracy did not differ between novice and experienced operators.

Some limitations must be acknowledged: first, only 17G needles were used. It has been suggested that liver deformation may vary depending on needle diameter and tip type^22^. Second, there was a lack of comparison with manual needle insertion. However, an accuracy of 4.53 mm for manual insertions has been reported elsewhere under similar conditions^12^. Third, we used general anesthesia and muscle relaxant because it was not possible to perform the procedure under local anesthesia in pigs. Thus, further studies are required to explore needle placement accuracy outside the context of general anesthesia. Fourth, no data were collected on radiation exposure. However, only minimal CT-scan acquisitions were performed (two for apnea repeatability and treatment planning, one for needle accuracy assessment and one for safety assessment), and no radiation was delivered to the operator. Despite the impossibility of performing intermediate scan without decoupling the needle from the robot, which might be regarded as a drawback, the reliability of the system allows the needle to be safely inserted in one shot. Finally, we found that accuracy did not differ between novice and experienced operators, but this may be due to the lack of statistical power caused by the limited number of needles inserted in this pilot study.

## Conclusion

Robotically-assisted needle insertion in the liver using the robotic device was shown feasible, safe and accurate in a swine liver model. Accuracy was influenced neither by the trajectory length nor by the trajectory angulation nor by the operator’s experience. Based on these encouraging in vivo results, a prospective human clinical trial is currently recruiting.

## Data Availability

The datasets used and/or analysed during the current study are available from the corresponding author on reasonable request unless our institutional review board disapproved.
