# Author Correction: Feasibility, safety and accuracy of a CT-guided robotic assistance for percutaneous needle placement in a swine liver model

**DOI:** 10.1038/s41598-021-87093-2

**Published:** 2021-04-09

**Authors:** Boris Guiu, Thierry De Baère, Guillaume Noel, Maxime Ronot

**Affiliations:** 1 Department of Radiology, St-Eloi University Hospital, 80 avenue Augustin Fliche, 34295 Montpellier, France; 2 Department of Interventional Radiology, Gustave Roussy Institut, Villejuif, France; 3 Department Pre‑Clinical, Biomedical and Analytical Investigations, Biovivo/Claude Bourgelat Institut, VetAgro Sup, Marcy l’Etoile, France; 4 Department of Radiology, Beaujon Hospital, APHP.Nord, Clichy, & Université de Paris, Paris, France

Correction to: *Scientific Reports* 10.1038/s41598-021-84878-3, published online 04 March 2021

The original version of this Article contained an error in the order of the Figures. Figure 4 was published as Figure 7, Figure 6 was published as Figure 4, and Figure 7 was published as Figure 6. The Figure legends were correct.

The original Figures 4, 6 and 7 and accompanying legends appear below.

**Figure 4:**
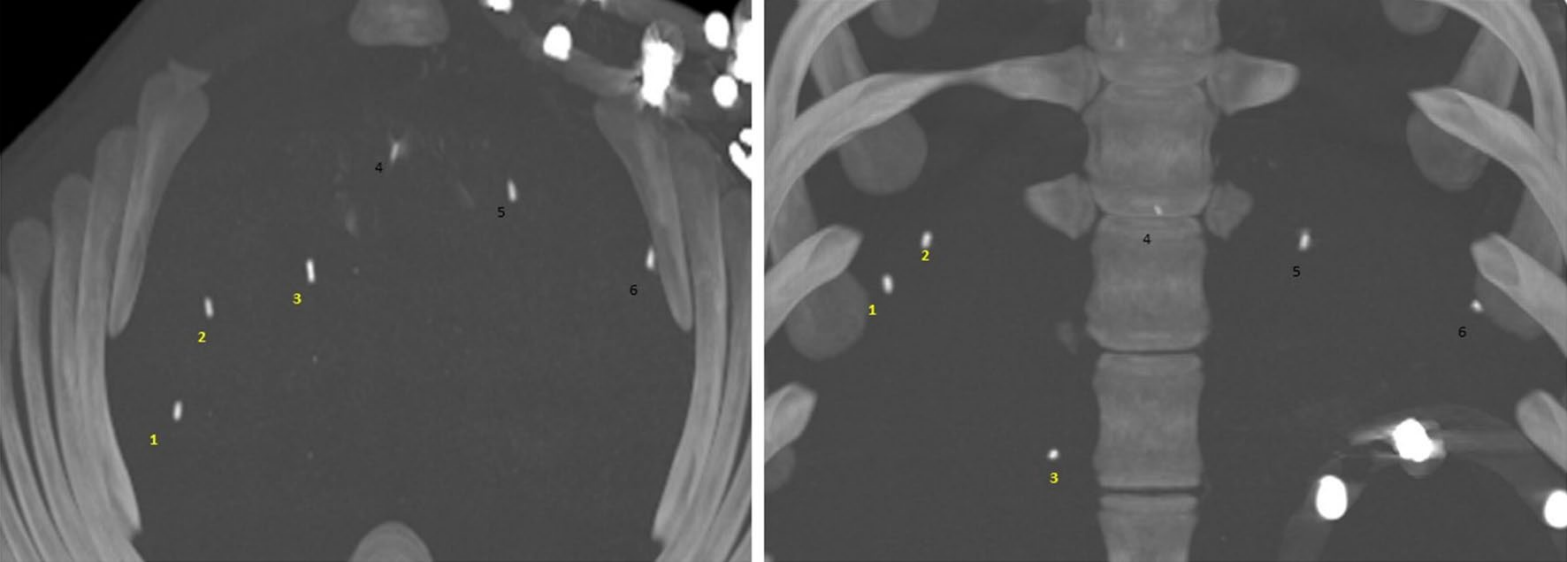
Screenshot of the planning software (home-made software). The needle trajectory (in yellow) is planned by defining the entry (skin) and the target points.

**Figure 6:**
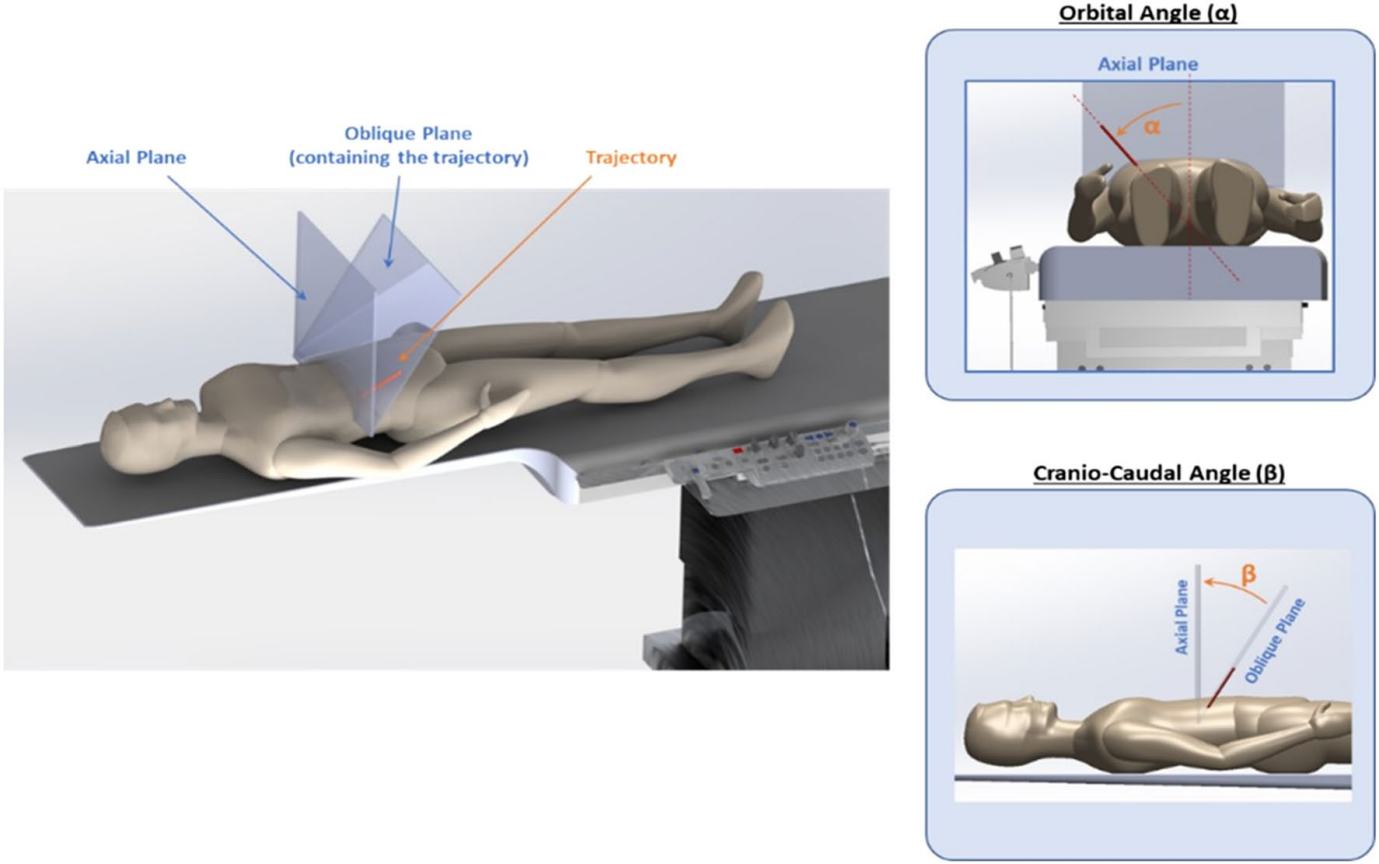
Axial and coronal maximum intensity projection CT images showing the placement of fiducials in a swine live.

**Figure 7:**
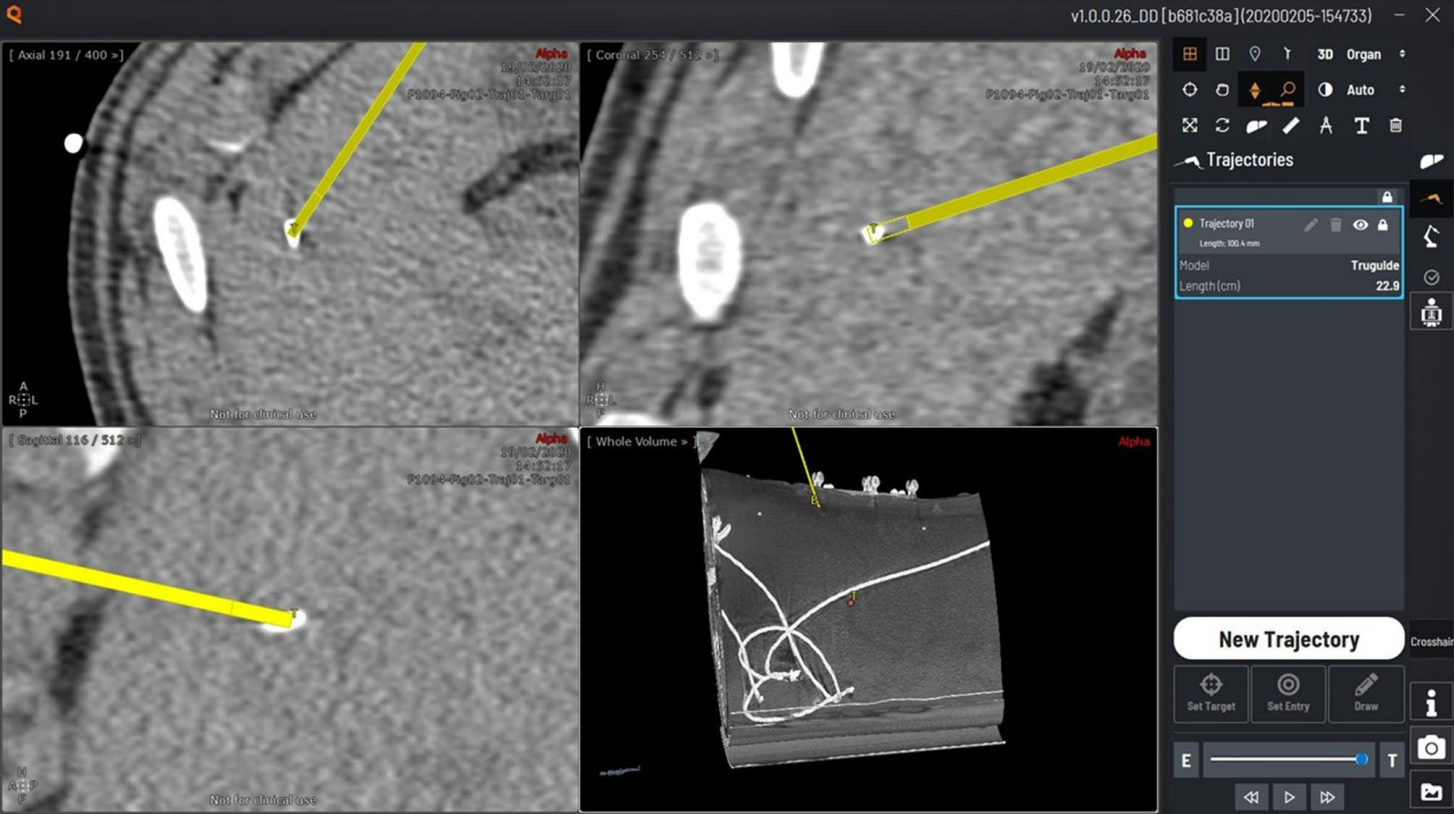
Definition of orbital and cranio-caudal angulations.

The original Article has been corrected.

